# Enhancing AI-based diabetic retinopathy diagnosis through universal cross-camera image adaptation

**DOI:** 10.1136/bmjophth-2025-002238

**Published:** 2025-11-21

**Authors:** Sanil Joseph, Xiaotian Chen, Chi Liu, Zhuoting Zhu, Kim Ramasamy, Thulasiraj D Ravilla, Zongyuan Ge, Mingguang He

**Affiliations:** 1 Ophthalmic Epidemiology, Centre for Eye Research Australia, East Melbourne, Victoria, Australia; 2 Department of Surgery (Ophthalmology), The University of Melbourne, Parkville, Victoria, Australia; 3 Lions Aravind Institute of Community Ophthalmology (LAICO), Aravind Eye Care System, Madurai, Tamilnadu, India; 4 Optain Health Pty Ltd, Melbourne, Victoria, Australia; 5 Faculty of Data Science, City University of Macau, Macao, China; 6 Vitreous and Retina Service, Aravind Eye Hospitals and Postgraduate Institute of Ophthalmology, Madurai, Tamil Nadu, India; 7 The AIM for Health Lab, Faculty of Information Technology, Monash University, Clayton, Victoria, Australia; 8 School of Optometry, The Hong Kong Polytechnic University, Kowloon, Hong Kong; 9 Research Centre for SHARP Vision (RCSV), The Hong Kong Polytechnic University, Kowloon, Hong Kong; 10 Centre for Eye and Vision Research (CEVR), Hong Kong Science Park, Hong Kong

**Keywords:** Imaging, Retina, Public health, Diagnostic tests/Investigation, Vision

## Abstract

**Objective:**

To evaluate the effectiveness of a deep learning-based style adaptation strategy in improving the diagnostic accuracy and cross-camera generalisability of artificial intelligence (AI) for detecting diabetic retinopathy (DR).

**Methods and analysis:**

This diagnostic study involved prospective recruitment of patients aged 50 years and older attending the outpatient clinic at a tertiary eye hospital in Southern India, between 14 June and 5 August 2022. Paired macula-centred retinal images were captured using two fundus cameras: Optain Resolve (portable, automated) and Topcon NW400 (static, manual). A style adaptation model, the Style-Consistent Retinal Image Transformation Network (SCR-Net), was applied to align image styles across cameras. The AI-based DR detection model, developed using the InceptionNeXt-T architecture, was trained on images from the EyePACS data set and evaluated under three scenarios: (1) training and testing on original images (2) training and testing on SCR-Net-adapted images; and (3) training on a combined (original+adapted) data set and testing on adapted images. Diagnostic accuracy and preservation of image quality were evaluated.

**Results:**

The mixed training/testing approach (scenario 3) achieved the highest diagnostic accuracy for Optain images at 79.2% (95% CI 75.9% to 82.6%) with a Cohen’s kappa of 0.893 (95% CI 0.867 to 0.917). Adapted images preserved critical diagnostic features (peak signal-to-noise ratio, 29.35; structural similarity index measure, 0.847). Style adaptation reduced false positives in Optain images while maintaining robust diagnostic performance for Topcon images, effectively addressing cross-camera variability.

**Conclusion:**

Style adaptation using SCR-Net enhances the consistency and generalisability of AI-based DR detection systems by reducing false positives and maintaining robust performance across camera systems. This approach has the potential to democratise access to early DR diagnosis in underserved regions. This study was conducted at a single centre using a limited set of fundus cameras, which may affect the generalisability. Nonetheless, further validation across diverse imaging systems and clinical settings is warranted to support broader applicability.

**WHAT IS ALREADY KNOWN ON THIS TOPIC**

- Artificial intelligence (AI)-based diabetic retinopathy (DR) detection is increasingly used in clinical practice, but their performance varies across images from different fundus cameras due to intercamera discrepancies. Style adaptation techniques have been explored to standardise imaging characteristics and improve AI generalisability.

**WHAT THIS STUDY ADDS**

- This study demonstrates that style adaptation using Style-Consistent Retinal Image Transformation Network model can improve diagnostic consistency of AI-based DR detection across different fundus camera systems. It minimises cross-camera variability and preserves diagnostic features without requiring prior access to source-style images, making it scalable and adaptable.

**HOW THIS STUDY MIGHT AFFECT RESEARCH, PRACTICE OR POLICY**

- These findings support the integration of portable fundus cameras into AI-driven DR screening programmes, potentially expanding access to automated diagnosis in diverse clinical and resource-limited settings. This approach may inform future policies on AI validation and deployment frameworks across heterogeneous imaging environments.

## Introduction

 Diabetic retinopathy (DR) is a major complication of diabetes mellitus and one of the leading causes of blindness in the working-age population worldwide.^1^,^3^ Early detection and timely intervention are crucial in preventing vision loss caused by DR.^4^ Traditional methods of DR diagnosis rely on retinal examination by trained specialists, which can be resource-intensive and limited in accessibility, especially in low-resource settings.^5 6^

Artificial intelligence (AI) has emerged as a promising tool for the automated detection of DR, leveraging deep learning algorithms to analyse retinal images and identify signs of the disease. Several studies have demonstrated the high accuracy of AI-based systems in DR diagnosis, which can potentially reduce the burden on healthcare systems and increase access to screening programmes.^7^,^10^ Despite these advancements, the effectiveness of AI models is often restricted by the diversity of the populations and the imaging systems used during training.^5 11^ Most AI algorithms for DR detection have been developed and validated using retinal images from specific populations and camera systems, limiting their generalisability and performance across different settings.^57^,^10^

The variability in image quality and characteristics across different fundus cameras poses a significant challenge to the universal application of AI models.^11 12^ Portable fundus cameras are increasingly used in diverse clinical settings due to their convenience and cost-effectiveness.^13 14^ However, discrepancies in the images captured by different fundus cameras—such as variation in resolution, field of view, image contrast and colour representation—can affect the performance of AI algorithms trained on data from a single camera type.^1 12^ For instance, differences in resolution and field of view may result in inconsistent visibility of retinal lesions, leading to misclassification or diagnostic errors. Variations in image contrast and colour representation can affect the detection of subtle features critical for identifying DR, such as microaneurysms or haemorrhages. These discrepancies introduce biases in the training process as the AI system tends to overfit to the characteristics of the source camera data (traditions desktop camera), reducing its ability to generalise to images from other devices. This underscores the importance of externally validating AI software on images acquired from different camera systems before clinical deployment.^15^ Addressing this challenge requires strategies that harmonise imaging output across devices, ensuring consistent features representation and enhancing the universal applicability of AI systems for retinal disease diagnosis.

Traditional style transformation approaches in AI diagnosis of retinal images have been employed to address intercamera variability, focusing on harmonising image features to improve model consistency and performance.^16^,^18^ These methods typically rely on having access to source-style images to train the transformation model, which can limit their scalability and applicability in settings where such data is unavailable. Generative adversarial networks (GANs), including models like CycleGAN, have been widely explored to standardise retinal images by aligning style features, such as illumination and texture, across diverse imaging systems.^19 20^ These methods leverage unpaired image-to-image translation to adapt the visual characteristics of images from one camera system to another, enabling better compatibility for AI analysis. Studies have demonstrated the utility of CycleGAN in medical imaging for harmonising image modalities such as MRI and CT, and in ophthalmology for adapting fundus images to improve segmentation or disease detection.^5 21^ However, traditional GAN-based style transfer models often require substantial computational resources and depend heavily on the availability of well-curated data sets to train reliable mappings. Moreover, they are inherently limited by their need for prior access to both source and target image styles during training, which restricts their scalability and adaptability in real-world scenarios where such paired data sets may not be available.^9 12 16^

In this context, we propose a universal adaptation model for AI-based DR diagnosis using image-to-image style transfer techniques. We trained a Style-Consistent Retinal Image Transformation Network (SCR-Net)^22^ model capable of standardising fundus image styles without requiring prior training on the source domain, making it a scalable and flexible solution for intercamera variability. Unlike traditional generative AI augmentation methods, SCR-Net overcomes key limitations: it does not require paired data sets from source and target styles, ensuring structural consistency without the need for extensive annotations or domain-specific data. By leveraging synthesised images to enforce style consistency and extracting high-frequency components (HFCs) to preserve critical retinal structures, SCR-Net ensures that essential diagnostic features are retained during style transformation. This addresses a common limitation of other methods, which often focus on visual style transformation but neglect the impact on clinical relevance. By adapting retinal images captured by a portable fundus camera to the style of images from a standard camera used in clinical practice, we aimed to enhance the diagnostic accuracy of the AI classification model. Therefore, the primary objective of this study was to evaluate the potential of cross-camera AI adaptation techniques in enhancing diagnostic accuracy across diverse populations.

## Materials and methods

We enrolled patients aged 50 years and older attending the outpatient clinic at Aravind Eye Hospital (AEH), a tertiary level eye hospital in Southern India. Patients were prospectively recruited and retinal images were captured between 14 June and 5 August 2022. We excluded patients with recent ocular surgeries or with eye infections. The study protocol was approved by the Institutional Review Board of AEH and adhered to the tenets of the Declaration of Helsinki. Informed consent was obtained from all participants before enrolment. Patients or members of the public were not involved in the design, conduct, reporting or dissemination plans of this research. The retinal images used in this study were captured from patients attending routine clinical care, but patient involvement was limited to participation and did not extend to research planning or implementation.

### Pairwise image acquisition

Paired retinal images were captured using two different fundus cameras: (1) a portable, automated fundus camera (Optain Resolve OPTFC01, Optain Health, Melbourne, Australia) featuring a compact design, autofocus capability and an 8-megapixel sensor, designed for ease of use in primary care settings, and (2) a static, manually operated fundus camera (Topcon NW400, Topcon Corporation, Tokyo, Japan) equipped with a higher-resolution 16-megapixel sensor, wide-field imaging and adjustable illumination settings, optimised for comprehensive retinal diagnostics in clinical environments. The Optain camera captured macula-centred images with a 50° field of view, while the Topcon camera provided a 45° field of view. One image from each eye of the enrolled patients was captured consecutively by each camera without mydriasis. The images were human-graded by a team of retina specialists, using the National Health Service (NHS) diabetic eye screening guidelines,^23^ to establish the reference standard for DR grading. Two retina specialists, masked to each other’s gradings, conducted the initial grading. Any disagreements were adjudicated by a senior retina specialist.

### Image transformation model

**Figure 1:**
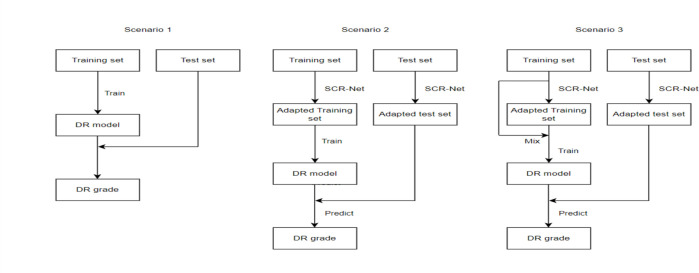
Overview of style alignment method using SCR-Net. SCR-Net, Style-Consistent Retinal Image Transformation Network.

We used an SCR-Net based image-enhancement model to align the style of fundus images captured by different cameras (figure 1). The model consists of three main components: a feature encoder (E), an HFC alignment decoder (DH) and an image restoration decoder (DR). The encoder extracts HFCs and latent features, which are passed to the decoders. The alignment decoder ensures structural consistency by aligning the extracted HFCs, while the restoration decoder reconstructs style-aligned images with preserved structural information. SCR-Net is optimised using a combination of alignment loss, restoration loss and cycle-consistency loss to achieve robust style transformation while preserving diagnostic details.

We trained the SCR-Net model using ∼20 000 desktop fundus camera images from the UK Biobank data set. For style alignment, fundus images from various cameras were processed through this model. The HFC extraction step preserves the structural details of the images, and subsequent reconstruction generates images with a specified style. This process transformed fundus images from different cameras into a uniform style suitable for AI analysis.

### AI-based deep learning model for DR detection

The development and validation of the AI algorithm used in this study has been described in detail in previous publications.^24^,^26^ The algorithm was trained on over 200 000 fundus photographs from the EyePACS data set, collected from diverse ophthalmic clinical settings. The deep learning model was developed using the InceptionNeXt-T architecture^27^ and included disease classification, image quality assessment and macular region detection. The model classified all images as ‘referrable DR’, ‘no DR’ or ‘ungradable’ categories. Referable DR was defined as more than mild NPDR (non-proliferative DR) and/or diabetic macular oedema according to the guidelines of the NHS diabetic eye screening.^28^

To evaluate the impact of style adaptation, the DR detection model was implemented under three distinct training scenarios. In each case, the model was trained using desktop fundus camera images from the EyePACS data set and tested on paired images from AEH captured by two different cameras in this study. All images were preprocessed by removing black background area and resizing to 512×512 pixels. The training configuration included the AdamW optimiser with a base learning rate of 1.0e-4 and a cross-entropy loss function. A series of experiments was conducted with different training data sets and configurations to optimise model performance in predicting Optain camera images.

### Evaluation of training scenarios

**Figure 2:**
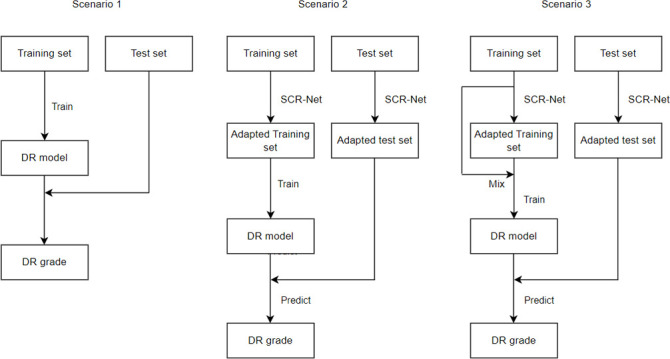
Flowcharts of scenarios 1–3. DR, diabetic retinopathy; SCR-Net, Style-Consistent Retinal Image Transformation Network.

We evaluated three training scenarios, using various combinations of original and transformed images, to assess the impact of style adaptation on model performance (figure 2). The model was trained using images from the EyePACS data set and tested using the paired images collected from AEH. In *scenario 1*, the deep learning model was trained on the original training set and tested on the original test set, serving as the baseline to assess model performance without any adaptation. In *scenario 2*, both the training and test sets were aligned using the style adaptation algorithm, with the model trained on the adapted training set evaluated on the adapted test set. In *scenario 3*, the original and SCR-Net-adapted training sets were combined (mixed) to train the DR model, which is then evaluated on the adapted test set. These three scenarios enabled a comprehensive evaluation of how style adaptation impacts model performance under different conditions.

### Evaluation metrics

To objectively evaluate the accuracy of the translated images, we performed a quantitative analysis using two widely recognised metrics: the structural similarity index measure (SSIM) and the peak signal-to-noise ratio (PSNR). SSIM quantifies the structural similarity between two images, where a value of 1 denotes perfect similarity and 0 indicates no similarity. PSNR assesses image quality by measuring the pixel-level differences between corresponding images, with higher values reflecting lower distortion.

### Statistical analysis

Metrics, including overall accuracy and Cohen’s kappa coefficient (along with its 95% CI), were used to evaluate the DR classification performance across different style alignment scenarios. Cohen’s kappa coefficient ranges from −1 to 1 and is commonly interpreted as follows: 0.40 to 0.60 indicates moderate agreement, 0.60 to 0.80 substantial agreement and 0.80 to 1.00 almost perfect agreement. Statistical analyses were performed using Python V.3.9.

## Results

We enrolled a total of 367 patients resulting in 734 pairs of images captured using the two fundus cameras. Two Topcon images were excluded due to poor quality, leaving 732 images for analysis. The mean (SD) age of participants was 54.6 (9.63) years, with 60.5% being male. Out of 732 image pairs, 555 pairs were gradable for both Topcon and Optain cameras.

### Style alignment performance

**Figure 3:**
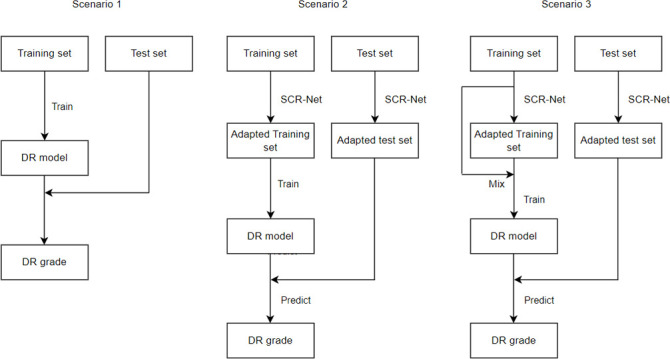
Examples of SCR-Net style alignment: source versus adapted fundus images. SCR-Net, Style-Consistent Retinal Image Transformation Network.

Figure 3 demonstrates the style alignment capabilities of SCR-Net for fundus images. The adapted images show consistent styles across different imaging systems while retaining essential anatomical and pathological details, confirming minimal information loss during the transformation process. The metrics for the transformed images, including PSNR and SSIM, were high, demonstrating strong similarity between original and transformed images. Optain images achieved a PSNR of 29.35 and an SSIM of 0.817, while Topcon images achieved a PSNR of 29.14 and an SSIM of 0.866.

### Confusion matrix analysis

**Figure 4:**
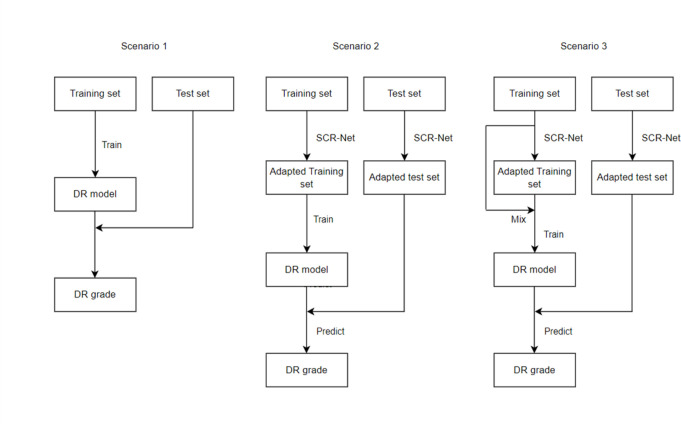
Confusion matrices of DR model performance across various scenarios. DR, diabetic retinopathy; PDR, proliferative diabetic retinopathy.

The confusion matrices in figure 4 reveal trends in DR classification performance for the Topcon and Optain images across the three scenarios. For Topcon images, the model performance was consistent across all scenarios. High accuracy was achieved in identifying ‘No DR’ cases, with stable sensitivity and specificity for advanced stages such as ‘Severe NPDR’ and ‘PDR’ (proliferative DR). This stability demonstrates that style alignment does not degrade performance for Topcon images.

For Optain images, *scenario 3,* using mixed images during training and transformed images during inference, achieved the best performance. *Scenario 1* showed high false positives for ‘No DR’ cases misclassified as ‘Moderate DR’. *Scenario 3* effectively reduced false positives, significantly improving specificity without compromising sensitivity for advanced DR stages. *Scenario 2* exhibited degraded performance, likely due to the limited diversity of image styles in the training set.

### Model performance metrics

Table 1 summarises the overall accuracy and Cohen’s kappa coefficients across scenarios. In *scenario 1* with both training and test styles set to the original images, Optain images demonstrated an accuracy of 75.9% (95% CI 72.3% to 79.4%) with a kappa of 0.863 (95% CI 0.836 to 0.889). For the Topcon images, accuracy was 77.8% (95% CI 74.4% to 81.3%), with a kappa of 0.883 (95% CI 0.843 to 0.913). In *scenario 2,* the performance on Optain images declined, with accuracy dropping to 69.7% (95% CI 65.9% to 73.6%) and the kappa coefficient falling to 0.823 (95% CI 0.789 to 0.855). In contrast, the Topcon-style images showed stable performance, with an accuracy of 78.2% (95% CI 74.8% to 81.6%) and a kappa coefficient of 0.895 (95% CI 0.863 to 0.921). In *Scenario 3*, where original and transformed images were mixed during training and transformed images were used for testing, the model achieved the best performance for Optain images, with accuracy improving to 79.2% (95% CI 75.9% to 82.6%) with a kappa to 0.893 (95% CI 0.867 to 0.917). Performance for Topcon images remained stable, with an accuracy of 79.1% (95% CI 75.7% to 82.5%) and a kappa of 0.892 (95% CI 0.859 to 0.922).

**Table 1:** Overall accuracy of Optain and Topcon images based on different scenarios

Scenario	Training style	Test style	Accuracy (95% CI)		Kappa	
			Optain	Topcon	Optain	Topcon
1	Original	Original	75.9% (72.3% to 79.4%)	77.8% (74.4% to 81.3%)	0.863 (0.836 to 0.888)	0.883 (0.844 to 0.913)
2	Transformed	Transformed	69.7% (65.9% to 73.6%)	78.2% (74.8% to 81.6%)	0.823 (0.789 to 0.855)	0.895 (0.863 to 0.921)
3	Mixed	Transformed	79.2% (75.9% to 82.6%)	79.1% (75.7% to 82.5%)	0.893 (0.867 to 0.917)	0.892 (0.859 to 0.922)

## Discussion

This study demonstrates the potential of deep learning-based image adaptation in addressing intercamera variability and enhancing AI diagnostic performance for DR. By leveraging mixed training data sets that include both original and style adapted images, the model achieved a modest yet consistent improvement in accuracy and generalisability, particularly for images from the novel portable imaging system (Optain), without compromising its performance on the original image style (Topcon). Notably, the style adaptation approach reduced false positives, preserved critical lesion details and minimised information loss during style transformation, thereby maintaining clinical relevance and diagnostic reliability.

The performance of AI models is significantly influenced by variation in imaging systems and populations used for training and testing. Differences in imaging quality, resolution and field of view affect how AI systems generalise across different data sets.^29^ Addressing these discrepancies often required significant data set augmentation or retraining on inputs from varied sources.^30^ AI models trained on homogeneous data sets often underperform when applied to data from diverse demographic or geographical populations. This limitation highlights the importance of incorporating multipopulation training data sets to enhance generalisability in real world settings.^31^ While previous approaches have focused on improving model robustness through larger databases or transfer learning, studies have shown that combining data sets from different sources can improve the robustness and generalisability of AI models.^32 33^

Our study introduces an innovative image adaptation strategy to address intercamera variability, leveraging style transfer previously applied in other medical imaging domains.^34 35^ By standardising retinal image styles, such methods effectively reduce discrepancy in image quality and appearance across different fundus cameras. Unlike previous methods, such as GANs,^19 20^ the proposed style alignment model does not require prior access to images from the source style for training, nor does it need to be trained separately for each source style. This flexibility is why it is referred to as a universal cross-camera image adaptation approach. This approach enables seamless integration of portable fundus cameras, such as Optain, into AI-based DR screening programmes without compromising diagnostic accuracy. With their affordability and compact design, portable fundus cameras are well-suited for deployment in remote and underserved areas where traditional imaging systems are often impractical.^11 36 37^ This advancement has the potential to significantly enhance access to early DR diagnosis in populations with limited access to conventional healthcare infrastructure.

### Strengths and limitations

A key strength of our study is the use of paired images from two distinct imaging systems, ensuring a robust evaluation of cross-camera performance. The inclusion of human-graded reference standards significantly enhances the reliability of the findings, providing clinically relevant benchmarks for DR classification. The systematic exploration of training and testing data sets—original versus adapted images—offers valuable insights into optimising AI performance across diverse imaging environments. Additionally, the study highlights the potential of style adaptation to address critical gaps in deploying AI across varied clinical and demographic settings. However, several limitations warrant attention. The single-centre recruitment design may limit the applicability of findings to broader populations. Moreover, the use of SCR-Net as a single style transfer model calls for validation with alternative models and imaging systems to confirm broader applicability. Another limitation is the lack of control over confounding factors such as operator skill and lighting conditions during image acquisition. Future studies should explore multicentre, multicamera data sets to assess the scalability of the proposed approach across diverse populations and imaging conditions. Investigating the integration of real-time style transfer during image acquisition is another promising avenue, which could streamline diagnostic workflows and improve operational efficiency.

Our finding underscores the transformative potential of style adaptations in bridging the gap between diverse imaging sources, paving the way for universal AI solutions in retinal disease detection. By enabling the seamless incorporation of portable fundus cameras into AI-driven diagnostic workflows, this approach holds immense promise for enhancing accessibility to high quality eye care, particularly in resource-limited settings. Such advancements are critical for scaling AI adoption in ophthalmology and ensuring equitable healthcare delivery globally.

## Data Availability

Data are available upon reasonable request.
